# Relationship Between Dietary Fiber and Vitamin C Intake and Oral Cancer

**DOI:** 10.3389/fpubh.2022.880506

**Published:** 2022-05-12

**Authors:** Jing Wang, Yi Fan, Jiawen Qian, Sijie Wang, Yanni Li, Mingming Xu, Fa Chen, Jing Wang, Yu Qiu, Lisong Lin, Baochang He, Fengqiong Liu

**Affiliations:** ^1^Department of Epidemiology and Health Statistics, Fujian Provincial Key Laboratory of Environment Factors and Cancer, School of Public Health, Fujian Medical University, Fuzhou, China; ^2^Key Laboratory of Ministry of Education for Gastrointestinal Cancer, Fujian Key Laboratory of Tumor Microbiology, Fujian Medical University, Fuzhou, China; ^3^Laboratory Center, The Major Subject of Environment and Health of Fujian Key Universities, School of Public Health, Fujian Medical University, Fuzhou, China; ^4^Department of Oral and Maxillofacial Surgery, The First Affiliated Hospital of Fujian Medical University, Fuzhou, China

**Keywords:** oral cancer, dietary fiber, vitamin C, food frequency questionnaires, case-control

## Abstract

**Background:**

Dietary fiber and vitamin C has been reported to play a possible role in tumorigenesis. However, few studies have estimated their association with oral cancer risk. In this project, we investigated the relationship between dietary fiber and vitamin C and oral cancer risk in adults in Southern China.

**Methods:**

382 patients newly diagnosed with oral cancer were matched to 382 hospital derived controls by frequency matching in age and sex. Pre-diagnostic consumption of dietary fiber and vitamin C intake were measured through food frequency questionnaire. Association between nutrients intake and oral cancer risk were evaluated by logistic regression. OR value and 95% confidence interval was calculated.

**Results:**

Intake of dietary fiber and vitamin C was significantly lower in oral cancer patients (8.15 g/day) than in control participants (8.88 g/day). Increased dietary fiber or vitamin C intake was linked to a decreased incidence of OC after adjustment of age, marital status, residence, BMI, occupation, education, tobacco smoking, alcohol consumption and family history of cancer *P*_*trend*_< 0.001). Compared with the lowest tertile, the adjusted OR of the top tertile of dietary fiber was 0.47 (95 % CI 0.32, 0.68). While the adjusted OR of the highest tertile was 0.60 (95 % CI 0.42, 0.87) compared with the lowest tertile of vitamin C.

**Conclusions:**

Dietary intake of fiber and vitamin C were lower in oral cancer patients than in control participants. Dietary fiber and vitamin C were inversely related to risk of oral cancer risk.

## Background

Oral cancer accounted for 40% of cancers of the head and neck, which includes squamous cell carcinomas of the tongue, floor of the mouth, buccal mucosa, lips, hard and soft palate, and gingival (1). At the same time, oral cancer has the characteristics of strong invasiveness and poor prognosis (2). There were 354,864 new cancer cases and 177,384 deaths per year worldwide, with a higher rate in developing countries (3–5). Smoking, alcohol consumption, poor dental hygiene, the human papillomavirus, and betel nut consumption are most commonly reported risk factors of oral cancer (6–12). However, in addition to the conventional risk factors described above, other environmental factors including dietary and nutrients have also been reported in recent research (13–15). The significance of vegetables and fruits abundant in dietary fiber and vitamins in carcinogenesis is garnering more attention among dietary factors (16, 17).

The findings of a significant systematic study released by the World Cancer Research Fund (WCRF) in 2019 indicated that adopting a diet rich in wholegrains, vegetables, fruit, and legumes may protect against cancer (18). As they include micronutrients with chemo preventive qualities, such as antioxidant activity, these foods are intended to help prevent certain malignancies. Vitamin C (L-ascorbic acid) is a water-soluble antioxidant that assumes a significant job in the body (19). A great volume of investigations have demonstrated that vitamin C possesses anti-oxidant, anti-inflammatory, and immunity-boosting qualities (19–21). In this proposal, dietary fiber, which is frequently acquired from grains, fruits, and vegetables, is also an essential element of a balanced meal. Dietary fiber was thought to be made up of polysaccharides with a high degree of polymerization that were resistant to digestion and absorption in the upper intestine and may subsequently be fermented in the gut (22), and have been linked to a lower risk of a range of tumors, including bladder cancer (23, 24), pancreatic cancer (25, 26), colorectal cancer (27–29), lung cancer (30, 31). However, evidence of a link between dietary fiber and head and neck cancers, notably oral cancer, is scarce.

In 2015 (32), the International Head and Neck Cancer Epidemiology (INHANCE) consortium reported that taking vitamin C from food can prevent oral cancer (32), and then in 2017 (33), they reported that taking more fiber might reduce the risk of HNC. These two studies, however, are mostly directed at whites and a limited number of Japanese, and there are very few publications on the connection between dietary fiber and the incidence of oral cancer in developing areas.

Thus the study was conducted to explore the potential link between dietary fiber and vitamin C consumption and the incidence of oral cancer in southern China. The aforementioned nutrients, we expected, would be inversely connected to the risk of oral cancer.

## Materials and Methods

### Study Population and Data Collection

A hospital-based case-control study was conducted in Fujian Province, China. From September 2016 to July 2020, oral cancer patients were recruited at the Department of Mouth & Maxillofacial Surgery, First Affiliated Hospital of Fujian Medical University. All patients were newly diagnosed primary oral cancer verified by histology. Patients with recurring or metastasized cancer, as well as those who had already had chemotherapy or radiation, were excluded. During the same time period, control subjects were selected from the same hospital's health examination center and were frequency matched to cases by age (10-year group) and sex. All subjects included were Chinese Han men and women between the ages of 18 and 80 who had resided in Fujian for at least 10 years.

In accordance with the Declaration of Helsinki, all subjects provided written informed permission. The investigation protocol was granted by the Institutional Review Board of Fujian Medical University (approval number: 2011053; approval date: March 10, 2011). Face-to-face interviews with study subject were performed by professional interviewers (unless the subject was too ill to respond). Finally, 382 oral patients and 382 control participants were included in the study.

### Dietary Assessment

Food frequency questionnaire was used to evaluate individuals' usual dietary habits during the previous year (FFQ). Majority food category include staple foods, beans and bean products, vegetables, fruits, animal foods, bacteria, algae, and nuts, beverages, and soups. The researchers gathered data on portion size and food consumption frequency. Subjects were informed food images to assist them measure their dietary consumption. By multiplying the intake frequency by the intake quantity and dividing it by 365 days, the average amount of each food item ingested (g/d) was determined. The Chinese Food Composition Tables (34) was used to determine dietary fiber (g/day) and vitamin C (mg/day) consumed.

### Statistical Analysis

For categorical variables, differences of demographic features and dietary data between cases and controls were assessed using the χ^2^-test or Fisher's exact test. The *t*-test was used to compare differences of continuous variables between the two groups when the data was normally distributed, and the Wilcoxon rank sum test was used when the data was skewed. Adjusted odds ratios (ORs) and the corresponding 95% confidence intervals (CIs) were determined with the lowest tertile of dietary fiber and vitamin C consumption as reference. The regression residual approach was used to adjust nutrient intake by total energy consumption. All of the data was analyzed using Stata software version 15.0. All *p*-values in this study are two-sided, and statistical significance was evaluated at the 0.05 level.

## Result

There were 764 individuals in total, of which 422 were men and 342 were women. Table 1 lists the demographic information of all participants in detail (Table 1). The median ages of the patient and control groups were 49 and 56 years, respectively. When compared to control people, the oral cancer group had a higher proportion of participants with alcohol consumption and a family history of cancer. The case and control groups had comparable distributions in ethnic group, education level, marital status, origin, BMI, smoking status, tea consumption, and employment.

**Table 1:** Main characteristics of case and control subjects.

**Variable**	**Case (%)** **(*n* = 382)**	**Control (%)** **(*n* = 382)**	**χ2**	***P*-value**
Age			2.755	0.097
<40	39 (10.21)	54 (14.14)		
≥40	343 (89.79)	328 (85.86)		
Sex			0.000	1.000
Male	211 (55.24)	211 (55.24)		
Female	171 (44.76)	171 (44.76)		
Education level			3.094	0.213
Illiterate	22 (5.76)	33 (8.64)		
Primary and middle school	244 (63.87)	226 (59.16)		
High school and above	116 (30.37)	123 (32.20)		
Marital status			2.861	0.091
Married	345 (90.31)	330 (86.39)		
Single	37 (9.69)	52 (13.61)		
Residence			2.235	0.135
Rural area	229 (59.95)	249 (65.18)		
Urban area	153 (40.05)	133 (34.82)		
BMI (kg/m2)			4.263	0.119
18.5–23.9	243 (63.61)	256 (67.02)		
<18.5	32 (8.38)	18 (4.71)		
≥24	107 (28.01)	108 (28.27)		
Smoking			1.9935	0.158
No	225 (58.90)	244 (63.87)		
Yes	157 (41.10)	138 (36.13)		
Alcohol consumption			7.323	0.007
No	250 (46.82)	284 (74.35)		
Yes	132 (57.39)	98 (25.65)		
Family history of cancer			16.323	<0.001
No	319 (83.51)	355 (92.93)		
Yes	63 (16.49)	27 (7.07)		
Occupation			1.743	0.418
Farmer	109 (28.53)	115 (30.10)		
Worker	45 (11.78)	55 (14.40)		
Staff and others	228 (59.69)	212 (55.50)		

**Figure 1:**
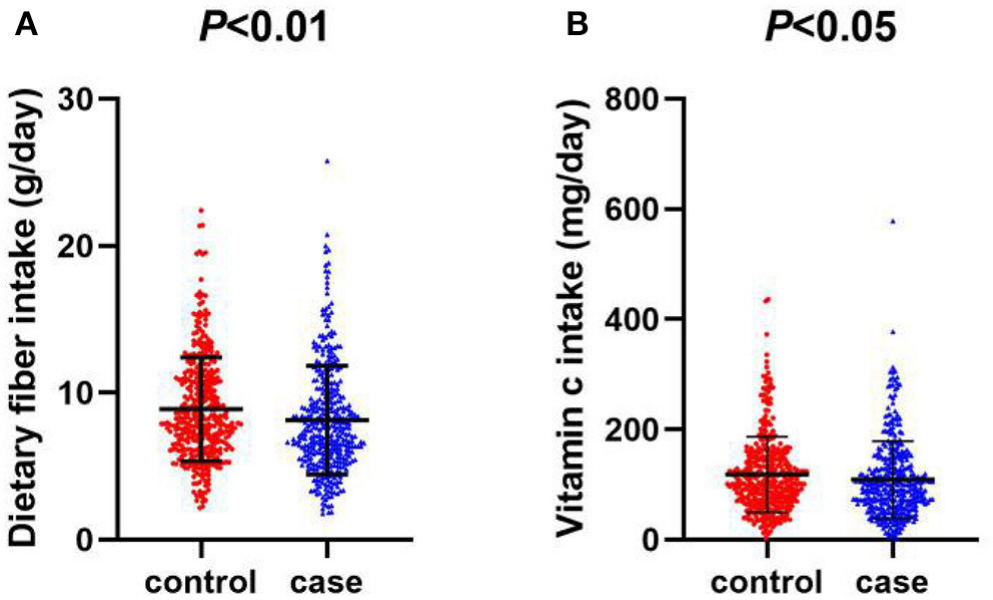
**(A)** Scatter diagram of daily intake of dietary fiber between oral cancer and control group. **(B)** Scatter diagram of daily intake of vitamin C between oral cancer and control group.

Compared with the controls, the intake of dietary fiber and vitamin C was significantly lower among cases (Figures 1A,B). The average daily intake of dietary fiber in the control group was 8.88 (g/day), while that in the case group was 8.15 (g/day). As for vitamin C, the average daily intake of the control group was 118.27 (mg/day), while that of the case group was 108.86 (mg/day). In the correlation analysis between daily average intake of dietary fiber, vitamin C and food groups, vegetables (r = 0.6560, *P* < 0.001) and fruits (r = 0.5463, *P* < 0.001) were the major source of dietary fiber, followed by soy foods, algae, nuts and grain, which are all related to dietary fiber intake, as data shown in Table 2. Vegetables (r = 0.8419, *P* < 0.001), fruits (r = 0.4022, *P* < 0.001), algae and nuts (r = 0.2052, *P* < 0.001) showed strong correlation with Vitamin C intake. Grains, soy food, meat and eggs, dairy, pickles and processed meat are not significantly related to vitamin C intake.

**Table 2:** Spearman correlation coefficient between daily average intake of dietary fiber and vitamin C and food groups in all study subjects.

	Dietary fiber^*^		**Vitamin C**	
	***r***	***P***	***r^**1**^***	***P***
Grains	0.1759	<0.001	0.0376	0.2999
Soy foods	0.3187	<0.001	0.1348	0.0002
Vegetables	0.656	<0.001	0.8419	<0.001
Fruits	0.5463	<0.001	0.4022	<0.001
Meat and eggs	.	.	0.0107	0.7677
Algae and nuts	0.2999	<0.001	0.2052	<0.001
Dairy	.	.	0.0481	0.1841
Pickle and processed meat	0.0096	0.7908	−0.0361	0.3189

* *The Spearman correlation coefficient between dietary fiber and vitamin C is 0.8091, P <0.001*.

Table 3 presents the odds ratio (OR) and 95% confidence interval (CI) for oral cancer across the tertile categories for the intake of dietary fiber and vitamin C. An inverse association between the intake of dietary fiber, vitamin C and risk of oral cancer was observed. In the crude mode, the ORs for the highest triplet compared with the lowest triplet intake were 0.52 (95% *CI*: 0.37–0.74; *P*_trend_ = 0.0006) for dietary fiber, and the ORs was 0.65 (95% *CI*: 0.46–0.92; *P*_trend_ = 0.0502) for vitamin C. After adjusting for age, marital status, residence, BMI, occupation and education, the individuals in the highest tertile of the intake of dietary fiber tended to have lower odds for oral cancer (*OR* = 0.51; 95% *CI*: 0.35–0.73; *P*_*trend*_ = 0.005) compared with those in the lowest tertile and a consistent result was observed in vitamin C (*OR* = 0.63; 95% *CI*: 0.44–0.90; *P*_trend_ = 0.0928). The result remained significant after further adjustment for tobacco smoking, alcohol consumption and family history of cancer, with *OR*s= 0.47 (95% *CI*: 0.32–0.68; *P*_*trend*_< 0.001) for dietary fiber, and 0.60 (95% *CI*: 0.42–0.87; *P*_*trend*_< 0.001) for vitamin C. Moreover, similarly association was observed when we treated the intake of dietary fiber and vitamin C as a continuous variable in the rude model, and adjusted models. The OR value of dietary fiber was 0.9337 (95% *CI*: 0.8950–0.9741, *P*_*trend*_< 0.001), while the OR value of vitamin C was 0.9974 (95% *CI*: 0.9952–0.9996, *P*_*trend*_< 0.001) in model 2.

**Table 3:** Association between dietary fiber and vitamin C intake and oral cancer by logistic regression.

	**T1**	**T2**	**T3**	***P_***trend***_***	**OR (95%CI)^†^**
**Dietary fiber**					
Crude	1.00 (ref.)	0.63 (0.45–0.89)	0.52 (0.37–0.74)	0.0006	0.9455 (0.9085–0.9840)
Model I OR (95%CI)	1.00 (ref.)	0.63 (0.45–0.89)	0.51 (0.35–0.73)	0.005	0.9410 (0.9034–0.9801)
Model II OR (95%CI)	1.00 (ref.)	0.60 (0.42–0.85)	0.47 (0.32–0.68)	<0.001	0.9337 (0.8950–0.9741)
**Vitamin C**					
Crude	1.00 (ref.)	0.80 (0.57–1.12)	0.65 (0.46–0.92)	0.0502	0.9980 (0.9959–1.0001)
Model I OR (95%CI)	1.00 (ref.)	0.79 (0.56–1.11)	0.63 (0.44–0.90)	0.0928	0.9978 (0.9957–0.9999)
Model II OR (95%CI)	1.00 (ref.)	0.78 (0.55–1.10)	0.60 (0.42–0.87)	<0.001	0.9974 (0.9952–0.9996)

*Mode I adjusted for demographic characteristics including age, marital status, residence, BMI, occupation, education*.

*Model II adjusted for demographic characteristics and tobacco smoking, drinking, family history of cancer*.

† *Dietary fiber and vitamin C intake treated as a continuous variable in the crude and two adjusted models*.

We also investigated whether the relationship between dietary fiber and vitamin C intake and oral cancer varies with different status of sex, smoking, alcohol consumption and family history of cancer (Tables 4, 5). The association between dietary fiber intake and oral cancer risk was more significant in female participants (*OR* = 0.43; 95% *CI*: 0.25–0.76; *P*_*trend*_< 0.001) and those without alcohol consumption (OR = 0.44, 95% *CI*: 0.21–0.90; *P*_*trend*_< 0.001). While association between intake of vitamin C and the risk of oral cancer remained significant in female (*OR* = 0.49, 95% *CI*: 0.28–0.85, *P*_*trend*_< 0.001) and those without alcohol consumption (*OR* = 0.55, 95% *CI*: 0.35–0.85; *P*_*trend*_< 0.001) compared with male and drinkers.

**Table 4:** Stratification analysis of dietary fiber intake and oral cancer.

**Variable**	**T1**			**T2**			**T3**			***P*_trend_**
	***n* (cases)**	***n* (controls)**	**OR**	***n* (cases)**	***n* (controls)**	**OR (95%CI)**	***n* (cases)**	***n* (controls)**	**OR (95%CI)**	
**Sex**										
Male	107	81	1.00	56	66	0.62 (0.38–1.00)	48	64	0.55 (0.34–0.91)	<0.001
Female	70	46	1.00	57	62	0.57 (0.34–0.98)	44	63	0.43 (0.25–0.76)	<0.001
**Smoking**										
No	90	76	1.00	75	88	0.66 (0.42–1.04)	60	80	0.55 (0.34–0.88)	<0.001
Yes	87	51	1.00	38	40	0.60 (0.33–1.09)	32	47	0.46 (0.25–0.85)	<0.001
**Alcohol consumption**										
No	94	85	1.00	87	98	0.61 (0.32–1.18)	69	101	0.44 (0.21–0.90)	<0.001
Yes	62	42	1.00	38	30	0.62 (0.40–0.94)	32	26	0.50 (0.33–0.78)	0.0002
**Family history of cancer**										
No	159	123	1.00	90	119	0.60 (0.42–0.86)	70	113	0.50 (0.34–0.73)	<0.001
Yes	18	4	1.00	23	9	0.41 (0.10–1.69)	22	14	0.36 (0.09–1.37)	0.002

**Table 5:** Stratification analysis of dietary vitamin C intake and oral cancer.

**Variable**	**T1**			**T2**			**T3**			***P*_trend_**
	***n* (cases)**	***n* (controls)**	**OR**	***n* (cases)**	***n* (controls)**	**OR (95%CI)**	***n* (cases)**	***n* (controls)**	**OR (95%CI)**	
**Sex**										
Male	98	84	1.00	57	66	0.74 (0.46–1.18)	56	61	0.76 (0.47–1.24)	0.0003
Female	58	43	1.00	68	62	0.76 (0.44–1.31)	45	66	0.49 (0.28–0.85)	<0.001
**Smoking**										
No	81	75	1.00	81	87	0.75 (0.47–1.19)	63	82	0.60 (0.37–0.97)	<0.001
Yes	75	52	1.00	44	41	0.77 (0.43–1.37)	38	45	0.67 (0.37–1.21)	<0.001
**Alcohol consumption**										
No	94	85	1.00	87	98	0.74 (0.48–1.13)	69	101	0.55 (0.35–0.85)	<0.001
Yes	62	42	1.00	38	30	0.85 (0.44–1.64)	32	26	0.82 (0.41–1.64)	0.0016
**Family history of cancer**										
No	140	122	1.00	102	119	0.77 (0.54–1.12)	77	114	0.61 (0.41–0.89)	0.0011
Yes	16	5	1.00	23	9	0.75 (0.19–3.04)	24	13	0.53 (0.15–1.96)	0.004

## Discussion

In this case-control designed study, we observed that higher dietary fiber and vitamin C consumption were related to a lower risk of oral cancer after adjustment of potential confounding factors. These findings are in accordance with the conclusions of two previous meta-analysis studies (32, 33).

Kawakita et al., etc., summarized and analyzed 10 case-control studies participating in the International Head and Neck Cancer Epidemiology consortium in a meta-analysis in 2017 (33), in which the majority of the participants were white and an inverse relationship between fiber consumption and oral cancer was identified. In a recent prospective research comprising 101,700 individuals, more than 70% of whom were white, they discovered that fiber consumption may protect against the formation of oral cancer (35). Meanwhile, in a prospective longitudinal cohort analysis of newly diagnosed patients with head and neck cancer, dietary fiber consumption was found to be adversely linked with all-cause mortality (36). And a study (35) reported the relationship between fiber intake and HNC risk by using data from screening tests for prostate cancer, lung cancer, colorectal cancer and ovarian cancer (PLCO). These findings back with previous research that suggests dietary fiber might reduce the incidence of HNC. There are relatively few reports on the relationship between dietary fiber consumption and cancer in developing area. A case-control study of 851 cases of nasopharyngeal carcinoma and 1,502 controls conducted in China (37) reported that dietary fiber intake was negatively correlated with the risk of nasopharyngeal carcinoma. Another study from China showed (38) that a high dietary fiber intake, particularly from vegetables and fruit, was negatively correlated to the incidence of colorectal cancer in Chinese people. Only one case-control study was carried out in Beijing in 1993 to report the relationship between dietary fiber and oral cancer (39), according which, fiber from fruits and vegetables has a substantial inverse connection with the likelihood of oral cavity cancer, whereas fiber from other sources does not. Our study from southeast China confirmed these findings, demonstrating that dietary fiber and vitamin C consumption were both negatively proportionate to the incidence of oral cancer.

Although further researches are needed to validate this conclusion, numerous molecular pathways have been postulated to explain why fiber consumption protects against oral cancer. Dietary fiber appears to have an anti-inflammatory effect (40, 41), through the creation of anti-proliferative and pro-apoptosis short-chain fatty acids by gut bacteria (42). By decreasing nuclear factor kappa-B activation and increasing anti-microbial peptide release, these short-chain fatty acids can lower pro-inflammatory cytokine expression (43, 44), whereas inflammation plays a key role in the etiology of precancerous lesions in the mouth (45, 46). Another possibility is that fiber-rich foods usually contain higher antioxidants, because higher fiber intake mostly origin from diet patterns rich in fruits, vegetables and beans (47). Likewise, dietary fiber may just be a sign of a balanced lifestyle in whole. The intake of dietary fiber has a substantial connection with the consumption of vitamin C (Table 2), according to our research. Vegetables and grains are the most abundant sources of dietary fiber, followed by fruits, algae, nuts and soybeans, which are all linked to dietary fiber consumption.

As for the relationship between vitamin C and cancer, many researchers have reported the beneficial effects of vitamin C on cancer (48–51) in both digestive tract cancer (52–55) and head and neck cancer (32, 56). In the Netherlands Cohort Study in 2015 (56), the incidence of HNC and HNC-subtypes was shown to be inversely related to vitamin C consumption. However, the results of a randomized double-blind RCT experiment in Japan in 2015 reported that vitamin C supplementation had no significance in preventing oral cancer (57). In this study, 46 Japanese participants with oral leukoplakia were randomly assigned to an experimental group and given low-dose β -carotene combined with vitamin C supplement to treat and prevent oral leukoplakia from malignant transformation. Two patients in the experimental arm and three in the control arm had oral cancer throughout the median 60-month follow-up period. Therefore, they claim that vitamin C cannot prevent oral cancer. However the number of subjects involved in this study and follow-up time are not justified enough to draw a definite conclusion. The inconsistent conclusions observed may attributed to variation in cancer types, research design, study population, sample size, methods of assessing dietary nutrition and confounding factors adjusted.

However, potential limitations of our study should also be notified. Firstly, we could not rule out the possibility of selection bias due to the hospital-based case-control study design, although the hospital covered most of the oral cancer cases from the selected area and we recruited control participant from the same hospital. Secondly, it was meaningful to study the relevance as a case-control study, but this information was not available. While the case and control participation rates were high, it is still possible that participating controls were more health conscious than the general population, thus biasing risk estimates away from the null. Thirdly, recall bias and measurement error in dietary assessment using an FFQ is difficult to avoid in a case-control study. Thus, the associations observed between dietary fiber, vitamin C and oral cancer should be interpreted cautiously.

In conclusion, the current investigation found that greater dietary fiber and vitamin C intakes were linked to a lower incidence of oral cancer in a southeastern Chinese population. Given the limitations of hospital-based case-control studies, further well-designed, particularly large-scale cohort studies, are needed to corroborate our findings.

## Data Availability Statement

The original contributions presented in the study are included in the article/supplementary material, further inquiries can be directed to the corresponding authors.

## Ethics Statement

The studies involving human participants were reviewed and approved by the Institutional Review Board of Fujian Medical University. The patients/participants provided their written informed consent to participate in this study.

## Author Contributions

JW (1st author), FL, and YF conceptualized the original idea for the study and have been involved in data collection, data analysis, and manuscript drafting. JQ assisted in statistical analysis. All authors have made substantial contributions to conception and design of the study, have read and approved the manuscript.

## Funding

This study was supported by Fujian Natural Science Foundation Program under Grant 2020J01639, Technology Development Fund from the Department of Education of Fujian Province Grant Number: 2019L3006 and Fujian Provincial Health Technology Project under Grant No. 2018-1-57.

## Conflict of Interest

The authors declare that the research was conducted in the absence of any commercial or financial relationships that could be construed as a potential conflict of interest.

## Publisher's Note

All claims expressed in this article are solely those of the authors and do not necessarily represent those of their affiliated organizations, or those of the publisher, the editors and the reviewers. Any product that may be evaluated in this article, or claim that may be made by its manufacturer, is not guaranteed or endorsed by the publisher.
